# Primary Hemiarthroplasty for the Treatment of Basicervical Femoral Neck Fractures

**DOI:** 10.7759/cureus.25210

**Published:** 2022-05-22

**Authors:** Ryan A Davis, Joseph D Henningsen, Scott Huff, Andrew D Schneider, Fady Y Hijji, Andrew Froehle, Indresh Venkatarayappa

**Affiliations:** 1 Orthopaedic Surgery, Wright State University, Dayton, USA; 2 Kinesiology and Health, Wright State University, Dayton, USA

**Keywords:** sliding hip screw, cephalomedullary nail, hip fracture, hemiarthroplasty, basicervical femoral neck

## Abstract

Purpose

Basicervical femoral neck fractures are uncommon injuries that occur at the extracapsular base of the femoral neck at its transition with the intertrochanteric line. Controversy remains in the orthopedic literature as to the optimal method of treatment for this fracture type given the inherent instability and greater rate of implant failure with traditional fixation constructs. The purpose of this study is to quantify the incidence and preferred treatment methods of basicervical hip fractures at a single, regional, Level 1 trauma center and to identify differences in postoperative complications between treatment options.

Methods

The present study is a retrospective case series from a single regional health network, including 316 patients with hip fractures. Basicervical femoral neck fractures were identified. Reoperation rates within 90 days, implant failures or nonunions, postoperative ambulation distances and range of motion, and discharge dispositions were compared across patients grouped by surgical treatment with either cephalomedullary nail, sliding hip screw, or hemiarthroplasty (HA).

Results

Basicervical femoral neck fractures represented 6.6% of this study population. The cephalomedullary nail group demonstrated rates of implant failure and return to the operating room within 90 days of 40% (4/10) and 20% (2/10), respectively. No patients who underwent hemiarthroplasty experienced a failure of fixation or return to the operating room.

Conclusions

This study suggests a much lower rate of fixation failure or need for reoperation with hemiarthroplasty treatment compared to cephalomedullary nail construct for basicervical femoral neck fractures and may be an underutilized treatment method for this fracture type. The promising results seen with this case series should encourage further investigation into HA as a primary treatment for these uncommon, yet challenging, fractures.

## Introduction

Hip fractures are a very common source of morbidity and mortality among the geriatric population. As our elderly population continues to live longer and have more active lifestyles, the incidence of hip fractures is estimated to continue to grow to affect over six million individuals globally by 2050 [1]. Hip fractures have been shown to be the most expensive osteoporotic fractures to treat from a healthcare and societal care perspective [2], thus it is critical for orthopedic surgeons to find ways to reduce the morbidity and mortality of surgical treatment [3].

Fractures about the hip are historically separated into two anatomic locations with respect to the location of the hip capsule in order to guide surgical treatment. Extracapsular fractures of the proximal femur involving the intertrochanteric region have traditionally been treated with sliding hip screw (SHS) or cephalomedullary nail (CMN) constructs to provide stability and compression across the fracture site necessary for anatomic union [4]. These treatment methods have been shown to be acceptable forms of fixation for the majority of extracapsular fracture variants [5]. Conversely, hemiarthroplasty (HA) is a common treatment method for displaced femoral neck fractures located within the articular capsule. This is due to the disruption of the tenuous vascular supply and the advanced rate of subsequent avascular necrosis of the femoral head with displaced fractures [6].

The basicervical region of the femoral neck represents the extracapsular base of the femoral neck at its transition with the intertrochanteric line. Basicervical femoral neck fractures are well-known for their inherent instability and greater rate of implant failure with traditional extracapsular fixation techniques [7]. This may be attributed in part to disruption of the posterior femoral calcar, which is integral in withstanding longitudinal loading forces in an axial direction but weak when subjected to tension or shear forces in the transverse plane [8].

Controversy remains in the orthopedic literature as to the optimal method of treatment for this fracture type [9-10]. The theoretical advantage of HA in the treatment of fractures of the basicervical region is the elimination of fracture displacement, screw cutout of the femoral head, nonunion, or femoral head avascular necrosis. Very little has been reported in the literature regarding the outcomes of HA for basicervical proximal femoral fractures, however, there is limited evidence to suggest lower reoperation rates compared to CMN or SHS constructs [11]. The purpose of this study was to quantify the incidence and preferred treatment methods of basicervical hip fractures at a single, regional, Level 1 trauma center and to identify any differences in postoperative complications or functional outcomes between treatment options. We hypothesized that the treatment of basicervical femoral neck fractures with HA would prove to be a safe treatment option that would result in fewer postoperative complications when compared with current treatment methods.

## Materials and methods

All study protocols were first reviewed and approved by the university’s institutional review board and approved by the committee on research ethics at our institution in accordance with the Declaration of the World Medical Association. Data were retrospectively extracted using Structured Query Language (SQL) programming similar to a previously published technique [12]. Briefly, a single regional health network’s electronic medical records (EMR; Hyperspace 2018; Epic Systems Corporation) were queried to identify surgically treated hip fractures from September 1, 2016, to August 31, 2018. Hip fracture patients were then further delineated based on the coded diagnosis. Diagnoses included for analysis were nonspecific hip fractures and basicervical femoral neck fractures. Any patients with a specific coded diagnosis of a subcapital femoral neck fracture or subtrochanteric or intertrochanteric femur fracture were excluded.

**Figure 1:**
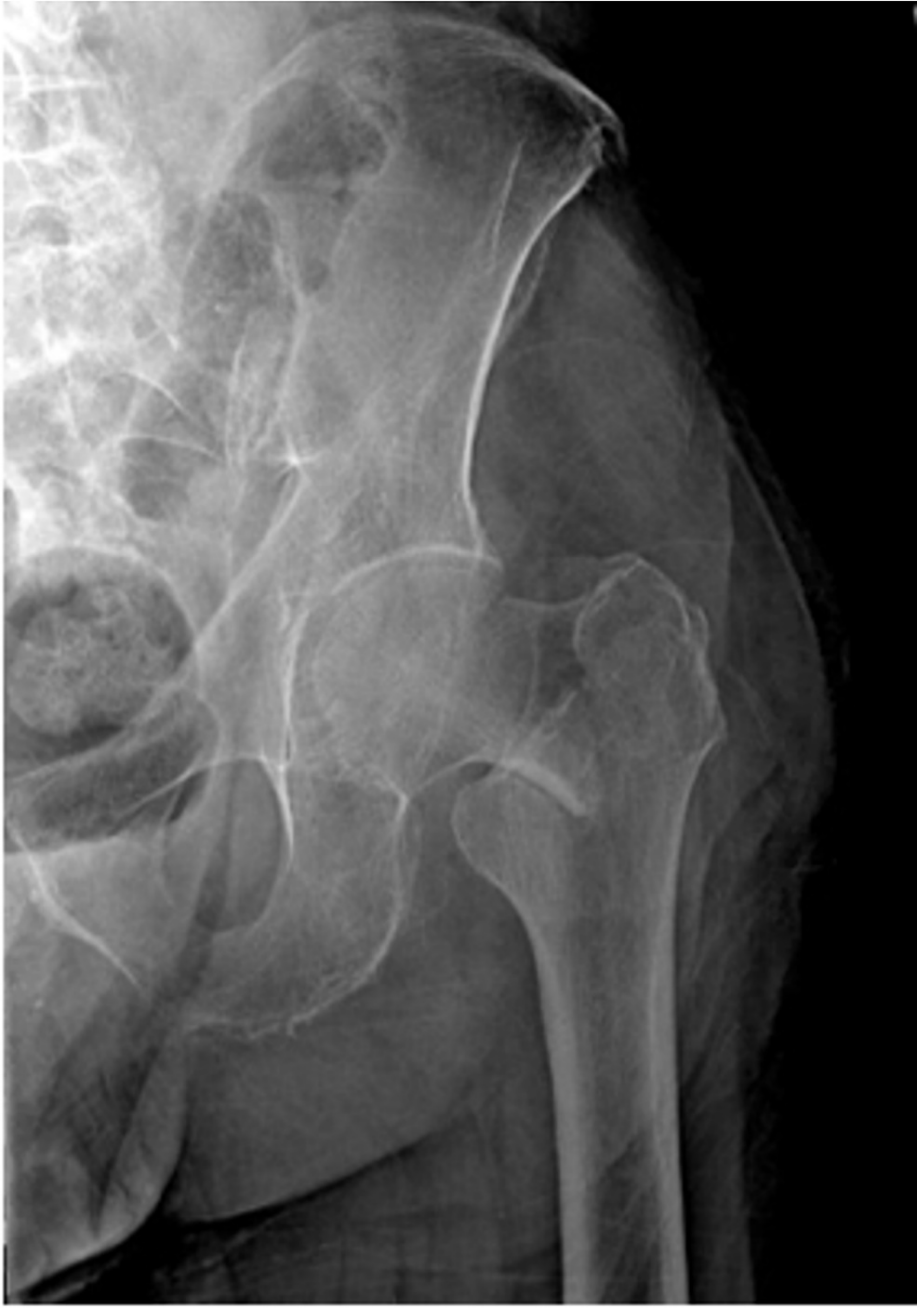
Radiograph demonstrating a basicervical femoral neck fracture

For each hip fracture patient that qualified, a manual chart review was performed to evaluate the injury fracture pattern. Basicervical femoral neck fractures were strictly defined as two-part fractures about the proximal femur, in which the fracture line was located at the base of the femoral neck at its junction with the intertrochanteric line (Figure 1). Fractures that resulted in the lesser trochanter becoming a separate fragment or the fracture line exiting distal to the lesser trochanter or out of the lateral cortex of the greater trochanter were not included in the study [9]. This definition aligns with the Orthopaedic Trauma Association classification of 31B3 for basicervical fractures of the proximal femur [13]. Images determined to meet the definition for basicervical fracture were first reviewed by an orthopedic surgery resident and then subsequently reviewed by two board-certified orthopedic surgeons, one specializing in trauma and one specializing in adult reconstruction. The patients that were felt to not accurately meet the definition for basicervical fracture were excluded from the study and the remainder of the patients were included for analysis.

**Figure 2:**
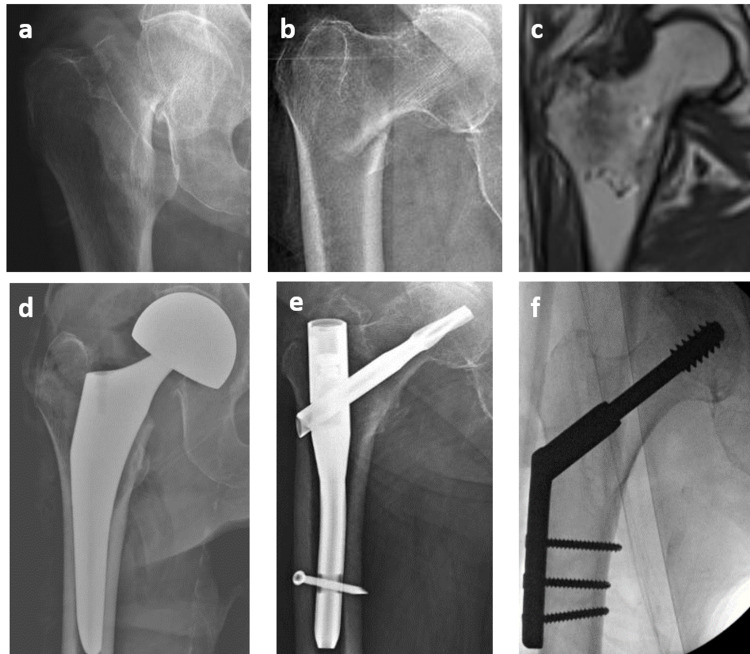
Radiographs of various peritrochanteric proximal femur fractures and their commonly performed surgical treatment methods 2-a and 2-b are radiographs of a displaced femoral neck fracture and a displaced intertrochanteric femur fracture, respectively. 2-c is an MRI of a nondisplaced intertrochanteric femur fracture. 2-d through 2-f are postoperative radiographs of the corresponding fractures above, demonstrating a hemiarthroplasty, a cephalomedullary nail, and a sliding hip screw, respectively.

Data for each patient identified as having a basicervical femoral neck fracture were then collected via database extraction and manual chart review. Preoperative data included general demographics, such as age, BMI, Charlson Comorbidity Index (CCI), and pre-injury functional status. Surgical treatments were stratified into CMN, SHS, and HA (Figure 2). Intraoperative fluoroscopic images were used to measure the tip-apex distance as described by Baumgaertner et al. [14]. The tip-apex distance is defined as the sum of the distance in millimeters from the tip of the lag screw to the apex of the femoral head on the anteroposterior radiograph and that distance as measured on a lateral radiograph. The images were first corrected for magnification and calibrated by using the known diameter and width of the implanted lag screw for all CMN and SHS constructs.

Complications were defined as mortality within one year, failure of the construct within one year defined by screw cutout, implant breakage, nonunion, or the need for revision surgery. Secondary outcomes included discharge disposition, postoperative range of motion, and postoperative ambulation distance.

Statistical analysis

Given the relative rarity of basicervical fractures, the sample size of patients with hip fractures and the resultant number of basicervical fractures in this study was intended to reflect similarly published literature regarding this fracture type [9,15]. Due to the relatively small sample size, there was insufficient statistical power to produce meaningful statistical tests of differences between treatment groups. Results are therefore reported as percentages for comparison between treatment modalities.

Note

The de-identified participant data for this manuscript are available upon request.

## Results

After the application of inclusion and exclusion criteria, 316 patients were identified for manual chart review. Twenty-one patients were identified as sustaining a basicervical femoral neck fracture, representing 6.6% of the initial sample. These basicervical fractures included in the study were operated on by a total of 12 surgeons, with the surgical treatment method being determined by surgeon preference and comfort level with the construct for treatment of the specific fracture pattern. Plain radiographs of the pelvis or femur were utilized in the initial diagnostic workup in all but one of the 21 basicervical cases, and computed tomography of the pelvis was additionally available for eight of these patients.

**Figure 3:**
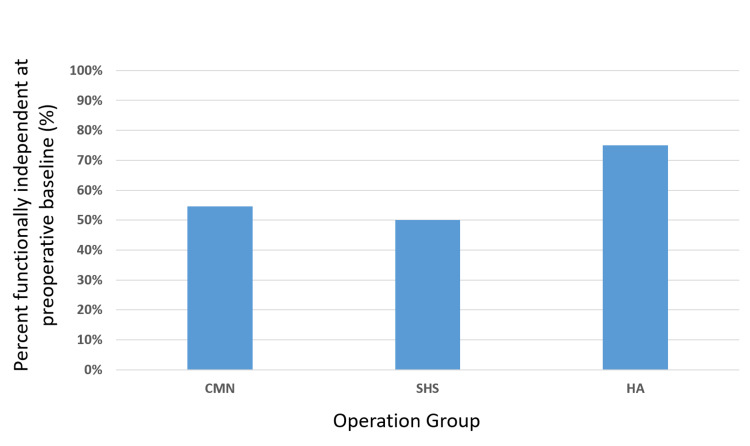
Bar graph illustrating data on the percentage of patients functionally independent at their preoperative baseline

**Figure 4:**
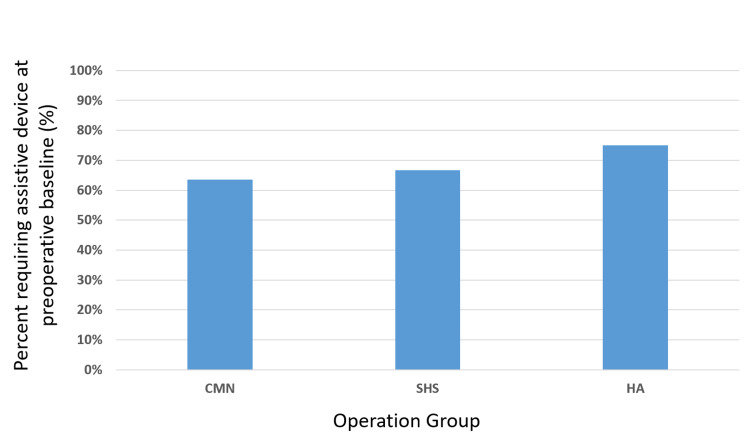
Bar graph illustrating the percentage of patients requiring some form of an assistive device at their preoperative baseline

The most common form of fixation was CMN (n=10), followed by SHS (n=7) and HA (n=4) (Table 1). Of the patients who underwent HA treatment, 75.0% were functionally independent at preoperative baseline as defined by the physical therapist, regardless of assistive device use (Figure 3). This frequency was appreciably higher than that of patients undergoing CMN or SHS (54.6% and 50.0%, respectively). Most patients across all three treatment methods were ambulatory with the use of some form of an assistive device at preoperative baseline, whether it was the intermittent use of a cane or reliance on a front-wheeled walker (percentage of patients using any assistive device at baseline; 63.6% CMN, 66.7% SHS, 75% HA) (Figure 4).

**Table 1:** Rate of basicervical fractures, stratification by implant type, and implant complications or failures *4/10 cephalomedullary nails (40%) experienced implant failure or nonunion. Two patients required a return to the operating room within 90 days

Total Number of Hip Fractures	316
Basicervical Hip Fractures	21 (6.6%)
Basicervical Fracture Treatment Method	
Cephalomedullary Nail	10
Sliding Hip Screw	7
Hemiarthroplasty	4
Complications or Implant Failure	
Cephalomedullary Nail	4*
Sliding Hip Screw	0
Hemiarthroplasty	0

**Figure 5:**
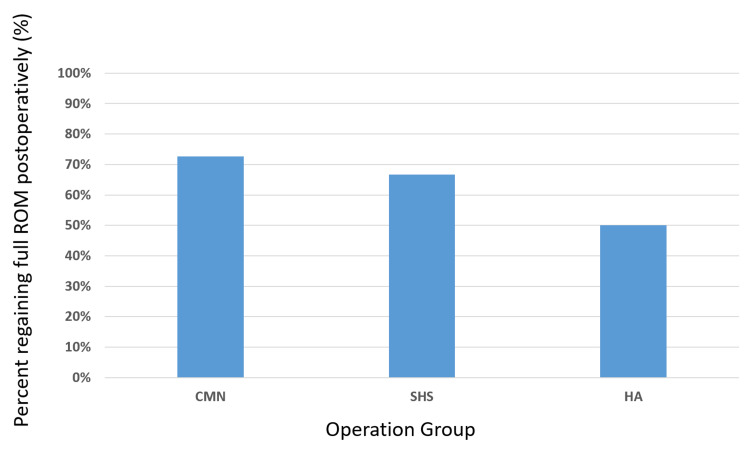
Bar graph illustrating the percentage of patients regaining their full range of motion (ROM) on the first postoperative physical therapy evaluation

Following surgery, the ambulation distance as recorded during the initial physical therapy evaluation appeared fairly uniform across all three treatment groups (median of 0 feet with each treatment method). Patients seem to regain a similar degree of baseline extremity range of motion during their initial physical therapy evaluation (50% HA, 72.7% CMN, 66.7% SHS), with a slightly greater percentage of patients undergoing CMN regaining motion compared to patients undergoing SHS or HA (Figure 5). More patients who underwent SHS fixation were discharged to a skilled nursing facility (83.3%) than those who were treated with CMN or HA (54.6% and 50%, respectively). The average tip-apex distance of all combined patients treated with CMN or SHS constructs was 17.7 mm, with the average tip-apex distance for patients treated with CMN or SHS constructs being 22.6 mm and 12.6 mm, respectively.

The CMN group was noted to have an implant failure rate of 40%, with the average tip-apex distance in the constructs that failed to be 20.9 mm. Of these patients who experienced a complication with their construct, one patient had a superior cutout of the femoral head compression screw and a resultant loss of construct fixation requiring a revision procedure. This patient subsequently went on to develop a postoperative infection of their revision procedure. The second patient sustained a non-traumatic fracture of the lateral cortex of the proximal femur with the associated collapse of the reduction one month after the initial surgery, requiring a revision procedure for stabilization of the fracture. The last two patients went on to nonunion at the femoral neck fracture site. Two of these patients returned to the operating room for revision surgery within 90 days at a rate of 20% (2/10). None of the patients treated with HA or SHS constructs experienced a failure of fixation or return to the operating room for any reason.

There were two patient deaths within one year of surgery; one patient who underwent HA died within 30 days of surgery of unknown causes while at an extended care facility, and one patient who underwent CMN died of bacterial pneumonia nine months postoperatively.

## Discussion

The optimal treatment method for basicervical femoral neck fractures remains a controversial topic in the orthopedic literature. The observed rate (6.6%) of basicervical fractures in the present study matches that of other studies and validates that these are uncommon injuries making confirmation of a reliable treatment option difficult [9,15]. In this case series, only four patients (19%) underwent HA for basicervical femoral neck fractures. Although it is difficult to extrapolate definitive conclusions from such small treatment groups, those fractures treated with HA exhibited no subsequent implant-related complications requiring a return to the OR and had functional data comparable to those fractures treated with SHS and CMN.

Several studies have demonstrated success with the use of SHS or CMN constructs when treating basicervical proximal femur fractures over more traditional intracapsular fixation methods. In a study by Sharma et al., 90 patients with basicervical femoral neck fractures were randomly assigned to treatment with cannulated cancellous lag screws, a dynamic hip screw with a derotational screw, or a proximal femoral nail [10]. The multiple cancellous lag screw construct was associated with the least stability during fracture healing and the longest mean time until fracture union. With the highest proportion of good-to-excellent Harris Hip Score results found among the SHS construct, they concluded that this was the optimal form of fixation among these three treatment methods. A cadaveric biomechanical model comparing SHS to multiple cancellous screws also favored fixation stability and strength towards the SHS [16]­. However, there are several studies that point toward the inadequacy of SHS for basicervical fractures. One such study by Lee et al. looked at the treatment of a basicervical femoral neck fractures with an extramedullary device such as an SHS and found this to be an independent risk factor for varus collapse at the fracture site and for fixation failure [17]. Kim and colleagues retrospectively looked at 106 patients with basicervical femoral neck fractures treated with osteosynthesis with either CMN or SHS and noted a higher rate of excessive fracture displacement when treated with SHS constructs [18]. Despite the absence of implant failure or the need to return to the operating room in the patients who underwent SHS in the present study, these previous reports should bring caution to its universal application for this unstable fracture pattern.

Proponents of treating basicervical femoral neck fractures with CMN argue that the intramedullary nature of the device allows for anatomic load sharing along the mechanical axis and can help buttress the medial femoral calcar [19]. Despite this theoretical mechanical advantage, a recent study by Watson et al. suggested that treatment of basicervical femoral neck fractures with CMN may be inadequate [9]. They performed a retrospective review of 246 peritrochanteric hip fractures treated with CMN and identified eleven patients with a two-part basicervical femoral neck fracture. Only five patients proceeded to heal their fracture without complications.

It has been widely accepted that the tip-apex distance defined in 1995 by Baumgaertner et al. has served as a predictor of lag screw cutout of the compression screw of the sliding hip screw construct [14]. Constructs with a tip-apex distance of <25 mm were found to be significantly less likely to fail. More recent studies have demonstrated that this same principle likely also applies to CMN constructs and confirms that the rate of compression screw cutout is much higher with greater tip-apex distances [20-21]. All of the patients in our study had a tip-apex distance of <25 mm, with an average of 17.7 mm. Of the four patients in the CMN group who experienced implant failure or nonunion, an average tip-apex distance of 20.9 mm was found. These results suggest that there are likely reasons outside of the accuracy of the lag screw placement or construct stability that contributes to the higher rate of implant failure seen in basicervical proximal femur fractures treated with CMN.

The present case series suggests that HA is a safe and effective method of treating basicervical femoral neck fractures, compared to CMN and SHS fixation methods. Lower revision rates in our cohort may be due to less reliance on patient bone quality for fracture fixation strength, which has been shown to be a risk factor for screw cutout and other means of fixation failure [22]. In addition, despite basicervical femoral neck fractures being extracapsular in nature, the risk of disruption to the vascular supply to the femoral neck and head may still be present and a risk factor for femoral head AVN or fracture nonunion. None of the patients in the present study who underwent HA experienced hip instability or dislocation despite the potential for a lower than normal femoral neck cut. In situations where there is a concern for inadequate metaphyseal support for an HA stem, the use of a diaphyseal fitting stem can be used to engage the femur in a more secure fashion.

We acknowledge that this study has limitations that must be mentioned. First, this is a retrospective study that is subject to biases from the inability to randomize patients to individual treatment methods. Second, the rarity of basicervical femoral neck fractures and overall low numbers leads to poor statistical power. This limits the generalizability of the conclusions. Finally, these fractures were treated by multiple different surgeons with individual preferences for certain fixation constructs and thus an implicit bias is present for which form of treatment was chosen. Thus, the results presented here should be interpreted as preliminary, but promising, meriting further study.

## Conclusions

In conclusion, this study suggests that HA may be an underutilized treatment method to address basicervical femoral neck fractures. The data presented show a lower rate of fixation failure or need for reoperation with HA treatment compared to CMN constructs. Initial functional recovery does not appear to vary significantly among the patients treated with any of the three treatment methods described. The promising results seen with this case series should prompt further investigation into HA as a treatment for these uncommon yet challenging fractures.
